# Exaggerated Ventilator-Induced Lung Injury in an Animal Model of Type 2 Diabetes Mellitus: A Randomized Experimental Study

**DOI:** 10.3389/fphys.2022.889032

**Published:** 2022-06-06

**Authors:** Álmos Schranc, Gergely H. Fodor, Roberta Südy, József Tolnai, Barna Babik, Ferenc Peták

**Affiliations:** ^1^ Department of Medical Physics and Informatics, University of Szeged, Szeged, Hungary; ^2^ Unit for Anesthesiological Investigations, Department of Acute Medicine, University of Geneva, Geneva, Switzerland; ^3^ Department of Anesthesiology and Intensive Therapy, University of Szeged, Szeged, Hungary

**Keywords:** mechanical ventilalion, metabolic disease, gas exchange, Ventilator Induced Lung Injury (VILI), lung tissue remodeling

## Abstract

Although ventilator-induced lung injury (VILI) often develops after prolonged mechanical ventilation in normal lungs, pulmonary disorders may aggravate the development of adverse symptoms. VILI exaggeration can be anticipated in type 2 diabetes mellitus (T2DM) due to its adverse pulmonary consequences. Therefore, we determined whether T2DM modulates VILI and evaluated how T2DM therapy affects adverse pulmonary changes. Rats were randomly assigned into the untreated T2DM group receiving low-dose streptozotocin with high-fat diet (T2DM, *n* = 8), T2DM group supplemented with metformin therapy (MET, *n* = 8), and control group (CTRL, *n* = 8). In each animal, VILI was induced by mechanical ventilation for 4 h with high tidal volume (23 ml/kg) and low positive end-expiratory pressure (0 cmH_2_O). Arterial and venous blood samples were analyzed to measure the arterial partial pressure of oxygen (PaO_2_), oxygen saturation (SaO_2_), and the intrapulmonary shunt fraction (Qs/Qt). Airway and respiratory tissue mechanics were evaluated by forced oscillations. Lung histology samples were analyzed to determine injury level. Significant worsening of VILI, in terms of PaO_2_, SaO_2_, and Qs/Qt, was observed in the T2DM group, without differences in the respiratory mechanics. These functional changes were also reflected in lung injury score. The MET group showed no difference compared with the CTRL group. Gas exchange impairment without significant mechanical changes suggests that untreated diabetes exaggerates VILI by augmenting the damage of the alveolar–capillary barrier. Controlled hyperglycemia with metformin may reduce the manifestations of respiratory defects during prolonged mechanical ventilation.

## Introduction

Volutrauma, barotrauma, and atelectrauma constitute the major pathophysiological background of ventilator-induced lung injury (VILI). The increased stress–strain initiates the overexpression of circulating proinflammatory mediators, thereby causing localized lung injury. This complex pathology is referred to as biotrauma (Curley et al., 2016) and develops frequently after prolonged mechanical ventilation. VILI is facilitated following long-term application of excessive tidal volume (VT), particularly without sufficiently high positive end-expiratory pressure (PEEP) (Smith et al., 2017).

Long-term hyperglycemia in untreated type 2 diabetes mellitus (T2DM) causes proinflammatory alterations of the endothelium, which entail endothelial and smooth muscle cell dysfunction with remodeling of the extracellular matrix (Nishizawa and Bornfeldt, 2012; Grinnan et al., 2016). Our previous studies demonstrated that chronic hyperglycemia also affects the respiratory system by reducing airway patency and compromising lung tissue viscoelasticity (Südy et al., 2020; Südy et al., 2021). In addition to these structural changes, T2DM induces persistent alveolar-capillary dysfunction due to the endothelial damage and/or surfactant depletion (Südy et al., 2020).

Lung diseases or comorbidities with pulmonary manifestations facilitate acute lung injury (ALI) and acute respiratory distress syndrome (ARDS) (Haberthür and Seeberger, 2016). Nevertheless, a potential of T2DM to protect against the development of ALI/ARDS has been reported in animal and clinical studies when sepsis was the primary cause of lung injury (Yu et al., 2013). The underlying mechanisms responsible for these seemingly controversial findings are not completely clear, with the involvement of the therapeutic management of T2DM was implicated (Tsaknis et al., 2012). While a different etiology of lung injury is frequently encountered after prolonged mechanical ventilation, the effect of T2DM on the severity of VILI has not yet been characterized, and modulation potential of T2DM treatment remains unknown.

Therefore, aimed at revealing whether T2DM modulates the development of adverse respiratory symptoms of VILI by characterizing the changes in the respiratory mechanics and gas exchange parameters. Because T2DM is treated with metformin as the first-line therapy, we also aimed at exploring the potential ability of this treatment to modify VILI in T2DM with controlled hyperglycemia.

## Materials and Methods

### Ethical Considerations

This randomized controlled study was approved by the National Food Chain Safety and Animal Health Directorate of Csongrád-Csanád County, Hungary (no. II./150/2020), on 18 March 2020. The study procedures were implemented in compliance with the guidelines of the Scientific Committee of Animal Experimentation of the Hungarian Academy of Sciences (updated Law and Regulations on Animal Protection: 40/2013. [II. 14.], the Government of Hungary) and the European Union Directive 2010/63/EU on the protection of animals used for scientific purposes. Results were reported according to the ARRIVE guidelines (Percie du Sert et al., 2020).

### Pretreatments and Group Allocations

Four-week-old male Wistar rats were randomly assigned into one of the following three groups: untreated T2DM model (T2DM, *n* = 10), metformin-treated T2DM model (MET, *n* = 10), and a control group (CTRL, *n* = 10). A well-validated T2DM model was adapted to induce diabetes in the T2DM and MET groups, which was based on feeding the animals with a special diet (HFHS U8954P Version0027, 30.3% fat, 18.4% protein, and 40.3% carbohydrate; SAFE^®^ Plant Diets & Custom Diets, Augy, France) from the age of 5 weeks. The validity and consistency of this model to reproduce the key features of diabetes have been demonstrated in our previous study (Südy et al., 2020). Rats in the CTRL group received a normal diet (A04, 3.1% fat, 16.1% protein, SAFE^®^ Plant Diets & Custom Diets, Augy, France) before the experiments. At the age of 7 weeks, rats in the MET and T2DM groups were treated with a single low-dose intraperitoneal injection of streptozotocin (STZ, 30 mg/kg) to reduce insulin production by the pancreas, whereas rats in the CTRL group received the vehicle (citrate buffer, pH 4.4) (Zhang et al., 2008; Skovsø, 2014). After 4 weeks, 300 mg kg^−1^·day^−1^ metformin (Merckformin 1,000 mg, Merck, Budapest, Hungary) in drinking water was administered to rats in the MET group (Garg et al., 2017; Liu et al., 2018). All rats were housed for 15 weeks before the experiments under close observation according to the animal welfare assessment and 3R guidelines.

To confirm the development of diabetes in the T2DM and MET groups, an intraperitoneal glucose tolerance test (IPGTT) was conducted 1 week after the STZ treatments. After a 12-h overnight fast and collection of baseline blood samples, a 2 g/kg 20% glucose solution was injected intraperitoneally. Next, blood samples were collected from the tail vein at 30, 60, and 120 min after the glucose injection to evaluate the changes in blood sugar levels (Accu-Chek Active blood glucose meter; Roche, Basel, Switzerland) (Antunes et al., 2016; Pilon et al., 2018). As an indirect marker of insulin resistance, the area under the curve (AUC) values were calculated from glucose levels (Wang et al., 2018). IPGTT was repeated 1 week before the experiments to confirm the development of controlled hyperglycemia in the MET group. Standard values were used to define diabetes, considering a fasting glucose level of 8.3 mmol/L and above and a significantly higher level in the STZ-injected animals as compared to the control animals and/or a 120-min serum glucose level of 11.1 mmol/L and above (Furman, 2015).

### Animal Preparation

After the 15-weeks pretreatment period, rats were anesthetized by an intraperitoneal injection of sodium pentobarbital (45 mg/kg; Sigma-Aldrich, Budapest, Hungary). An 18G cannula was inserted into the trachea by tracheostomy under local anesthesia (subcutaneous lidocaine, 2–4 mg/kg), and it was then connected to a small animal ventilator (Model 683; Harvard Apparatus, South Natick, MA, United States). We applied volume-controlled mechanical ventilation (55–60 breaths/min, VT: 7 ml/kg, with a fraction of inspired oxygen of 21%). The femoral artery and vein were cannulated for drug administration, blood pressure measurements, and blood sample collection. Anesthesia was maintained using sodium pentobarbital (5 mg/kg, intravenous [iv], every 30 min). Rats were placed on a heating pad (model 507223F; Harvard Apparatus, Holliston, MA, United States), and the body temperature was maintained within the 37 ± 0.5°C range. Muscle relaxation was provided by neuromuscular blockade with repeated iv administration of pipecuronium (0.1 mg/kg every 30 min; Arduan, Richter-Gedeon, Budapest, Hungary).

### Blood Gas Measurements and Calculation of Intrapulmonary Shunt Fraction

For blood gas analysis, 0.15-ml samples of arterial and venous blood were collected simultaneously. The arterial partial pressure of oxygen (PaO_2_) and the arterial oxygen saturation (SaO_2_) were determined using a point-of-care blood analyzer system (Epoc Reader and Host; Epocal, Inc., Ottawa, ON, Canada). The capillary (CcO_2_), arterial (CaO_2_), and venous (CvO_2_) oxygen contents were calculated from the blood gas values and were used to determine the intrapulmonary shunt fraction (Qs/Qt) by applying the following modified Berggren equation (Berggren, 1964; Wagner, 2015):

$$\frac{Q_{s}}{Q_{t}}=\frac{CcO_{2}-CaO_{2}}{CcO_{2}-CvO_{2}}$$

### Airway and Respiratory Tissue Mechanics

A previously described wave tube model of the forced oscillation technique was used to determine the input impedance of the total respiratory system (Zrs) (Peták et al., 1997). The tracheal cannula was connected to a loudspeaker-in-box system, and the ventilation was suspended for a short period (8 s) at end-expiration. A small-amplitude pseudorandom signal (<1.5 cmH_2_O, with 23 non-integer multiple-frequency components between 0.5 and 20.75 Hz) was applied to the tracheal cannula through a wave tube (polyethylene; length 100 cm, internal diameter 2 mm). During the 8-s measurement periods, pressures were measured simultaneously at the loudspeaker and tracheal ends of the wave tube using miniature differential pressure transducers (Honeywell Differential Pressure Sensor model 24PCEFA6D; Honeywell, Charlotte, NC, United States). Zrs was calculated as the load impedance of the wave tube (Peták et al., 1997). At least four technically acceptable measurements were made at each stage of the protocol. The mechanical properties of the respiratory system were characterized by fitting a well-validated constant-phase model (Hantos et al., 1992) to the ensemble-averaged Zrs spectra. The model comprises the frequency-independent airway resistance (Raw) and airway inertance in series with a viscoelastic constant-phase tissue unit that incorporates tissue damping (G) and elastance (H) (Hantos et al., 1992).

### Lung Tissue Histology and Immunohistochemistry

After the completion of the experimental protocol, the animals were euthanized by an overdose of pentobarbital, and then a midline thoracotomy was performed. The lungs were fixed by introducing 4% paraformaldehyde (PFA) through the tracheal cannula at a hydrostatic pressure of 20 cmH_2_O. The lungs were removed in one piece from the thoracic cavity and stored in 4% PFA at 4°C overnight and then in phosphate-buffered saline solution. The tissue samples were embedded in paraffin, and 7-µm-thick sections were cut using a microtome (Leica RM2521 RTS, Leica Microsystems GmbH, Wetzlar, Germany). The lung injury was determined by observing the sections stained with hematoxylin and eosin under a light microscope. Each slide was evaluated by three separate investigators in a blinded manner. To calculate the lung injury score (LIS), a total of 25 alveoli were counted in each field at ×400 magnification. Points were assigned according to previously established criteria. LIS was calculated using the following formula: LIS = [(alveolar hemorrhage points) + 2 × (alveolar infiltrate points) + 3 × (fibrin points) + (alveolar septal congestion points)]/total number of alveoli counted in the field (Matute-Bello et al., 2001). To evaluate oxidative DNA damage, sections were selected for the permanent immunocytochemical staining of 8-hydroxy-2ʹ-deoxyguanosine (8-OHDG). After routine deparaffinization, heat-induced antigen retrieval was performed by immersing the slides in 0.01 M sodium citrate buffer (pH 6.0). Endogenous peroxidases were blocked using 5% H_2_O_2_. Nonspecific protein-binding sites were blocked using 5% normal goat serum (Merck, Kenilworth, NJ, United States), and the sections were permeabilized with 0.5% Triton X-100 (Merck, Kenilworth, NJ, United States) in Tris-buffered saline. The sections were incubated overnight with mouse monoclonal anti-8-OHDG (Abcam, ab48508, 1:200) at 4°C. The subsequent steps of incubation consisted of an enhancer reagent at room temperature for 1 h and horseradish-peroxidase-linked secondary antibody also at room temperature for 3 h. These are the components of the Polink-2 Plus HRP Detection Kit (for mouse primary antibody with diaminobenzidine (DAB) chromogen, D37-18, GBI Labs, Bothell, WA, United States). Positivity to 8-OHDG was visualized with DAB, and the sections were overstained with hematoxylin (Chen et al., 2018a). On each section, nine fields were digitally recorded using a Nikon-DS Fi3 camera attached to a Leica DM 2,000 Led light microscope (Leica Microsystems GmbH, Germany) at × 200 magnification. Fields were evaluated by manual cell counting using the Cell Counter plugin of ImageJ (Wayne Rasband, NIH) by two observers blinded to the experimental design (Schindelin et al., 2012).

### Experimental Protocol

**FIGURE 1:**
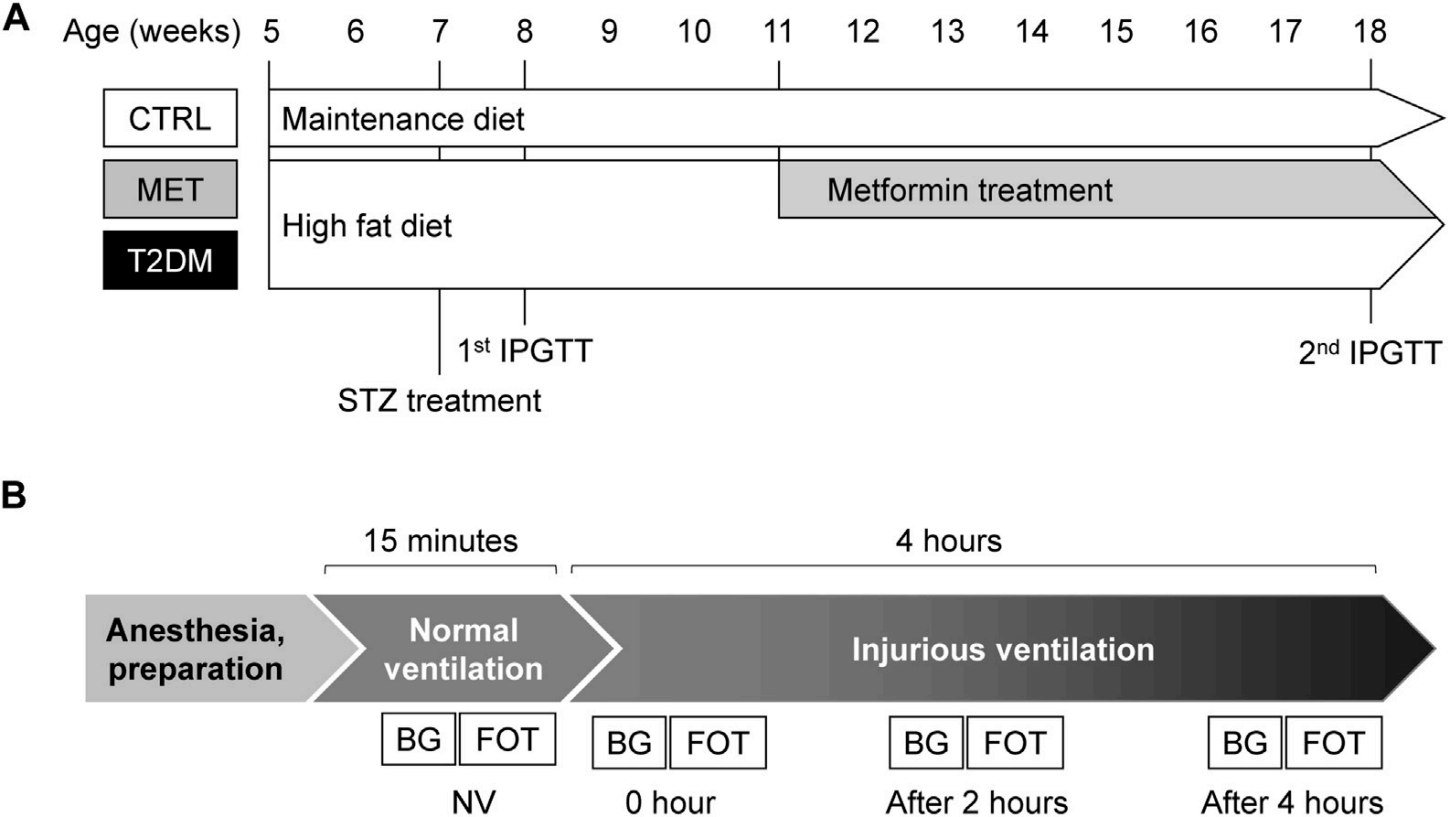
Schemes summarizing the pretreatment period **(A)** and the experimental protocol **(B)**. Panel **(A)** *:* Five-week-old rats were fed with maintenance (Group CTRL) or high-fat diet (Groups MET and T2DM) for 13 weeks. To model peripheric insulin resistance by lowering the serum insulin level, intraperitoneal streptozotocin (STZ) was used in the diabetic groups. A week after the injection intraperitoneal glucose tolerance test (IPGTT) was performed to confirm diabetes. Metformin treatment was initiated at the age of 11 weeks for animals in the MET group. The IPGTT was repeated at the end of the pretreatment period to confirm the controlled hyperglycemia in the MET group. Panel **(B)** After the 15-weeks pretreatment period, experiments were conducted under general anesthesia. The mechanical ventilation was initially performed with physiological parameters (NV: 7 ml/kg, PEEP: 3 cmH_2_O and 55–60/min frequency), followed by injurious ventilation (23 ml/kg, PEEP: 0 cmH_2_O and 25–30/min frequency). The forced oscillation measurements (FOT) and blood gas analyzes were (BG) performed four times, viz., at the end of the NV period and 0, 2, and 4 h during injurious ventilation. Abbreviations: CTRL, control group; MET, metformin-treated model of type 2 diabetes; T2DM, untreated model of type 2 diabetes; STZ, streptozotocin; IPGTT, intraperitoneal glucose tolerance test; NV, volume-controlled ventilation with physiological parameters; FOT, forced oscillation measurements; BG, blood gas analysis.

The experimental protocol is summarized in Figure 1. After the pretreatment period, the rats were anesthetized and ventilated in the volume control mode with physiological parameters (VT: 7 ml/kg, PEEP: 3 cmH_2_O and 55–60/min frequency) for 20 min. Airway opening pressure was monitored to evaluate the peak inspiratory pressure (PIP), and electrocardiogram and systemic blood pressure were also registered (Biopac, Goleta, CA, United States). Next, forced oscillatory measurements were performed to evaluate the respiratory mechanics, and then arterial and venous blood samples were collected for blood gas analyses. An injurious ventilation strategy was initiated to induce VILI by setting high VT (23 ml/kg) and low PEEP (0 cmH_2_O), and it was maintained for 4 h. To avoid severe hypocapnia, the ventilation frequency was reduced to 25–30/min to maintain minute ventilation and the end-tidal CO_2_ in the range of 25–30 mmHg. This ventilation has been demonstrated to induce VILI consistently by the simultaneous induction of barotrauma due to the high VT and atelectrauma as a consequence of low PEEP (Villar et al., 2009). Forced oscillatory measurement of the respiratory mechanics and blood gas analyses were conducted after a 15-min period that was allowed for the stabilization of respiratory and hemodynamic variables (0 h) and 2 and 4 h after the onset of injurious ventilation. After the completion of the measurement protocol, the animals were euthanized by an overdose of pentobarbital (200 mg/kg), and the lungs were removed for histological analysis, as detailed earlier.

### Exclusion Criteria

Thirty young male Wistar rats were enrolled in the study. Two animals in the MET group were sacrificed 12 h after the STZ treatment due to their unsatisfactory health condition. Due to technical issues with equipment and monitoring, we had to exclude one CTRL animal. We excluded three animals (two from the T2DM and one from the CTRL group) from the final analysis, due to circulatory destabilization and critical hypotension in the last period of injurious ventilation. Thus, 24 animals (CTRL, *n* = 8; MET, *n* = 8; T2DM, *n* = 8) were included in the final analysis.

### Data Analysis

Data are expressed as boxplots and mean ± standard deviation (SD). The Shapiro–Wilk test was used to evaluate data distributions for normality. Two-way repeated-measures ANOVA with Holm–Šidák post hoc analyses was used to explore the effects of diabetes induction and metformin therapy. One-way ANOVA with Holm–Šidák post hoc tests were applied to determine the differences in body weights and the AUC values of IPGTT and in the histological outcomes between the protocol groups. The oxygenation and respiratory mechanical parameters are represented either as absolute values, where two-way repeated-measures ANOVA with Holm–Šidák post hoc analyses was used to determine the changes during the injurious ventilation, or as relative values (difference between 0 and 4 h), where one-way ANOVA with the Holm–Šidák method was used. PaO_2_ was considered as the primary outcome variable to estimate sample size for repeated-measures ANOVA, with a power of 0.8 and an alpha of 0.05. This analysis showed that at least eight animals were required in each protocol group to detect a 15% statistically significant difference in the primary outcome (Bausell and Li, 2002). To account for potential drop-out, 30 animals (ten in each protocol group) were enrolled in the present study. Statistical analyses were conducted with a significance level of *p* < 0.05.

## Results

**FIGURE 2:**
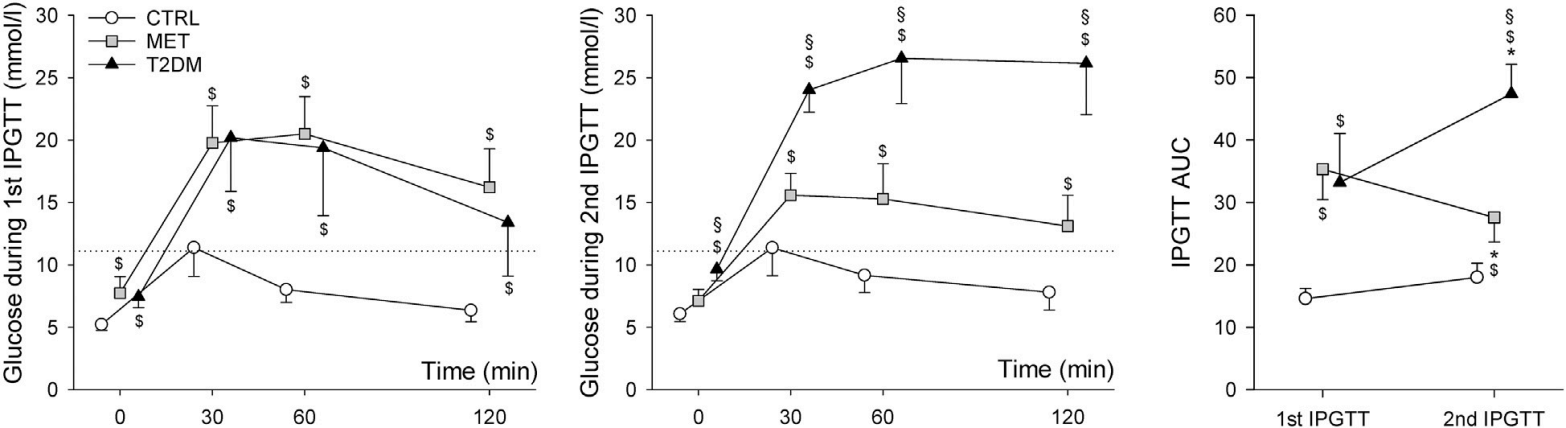
Blood glucose levels and the corresponding AUC values belonging to the 1^st^ and 2^nd^ IPGTT measurements during the pretreatment period. *p < 0.05 *vs*. 1^st^ IPGTT; $*p* < 0.05 *vs*. CTRL; §*p* < 0.05 *vs*. MET. Abbreviations: CTRL, control group; MET, metformin-treated model of type 2 diabetes; T2DM, untreated model of type 2 diabetes; IPGTT, intraperitoneal glucose tolerance test. Dotted line denotes the threshold glucose levels for 120-min blood glucose measurements (11.1 mmol/L).

The serum glucose levels and the corresponding AUC values during IPGTTs are summarized in Figure 2. The first panel summarizes the results of the 1^st^ IPGTT one week following the STZ injection. Although at that time the fasting glucose levels of the animals in the MET and T2DM groups did not consistently reach the threshold level of 8.3 mmol/L during the first tolerance test, significantly higher values were registered in both groups (7.7 ± 1.3 mmol/L and 7.4 ± 0.8 mmol/L, respectively) compared to the animals of the CTRL group (5.2 ± 0.4 mmol/L, *p* < 0.05 for both). However, the 120-min serum blood glucose values in the CTRL group (6.4 ± 0.9 mmol/L) were consistently below 11.1 mmol/L, with the MET and T2DM groups both exceeding this critical level (16.2 ± 3.1 mmol/L; 13.4 ± 4.2 mmol/L). This difference between the groups was statistically significant (*p* < 0.01). During the 2^nd^ IPGTT, the fasting glucose levels of CTRL and MET groups (6.1 ± 0.6 mmol/L and 7.1 ± 4.2 mmol/L, respectively) were in the normal range, but the values of T2DM animals (9.6 ± 0.9 mmol/L) were over the threshold level of 8.3 mmol/L. At 120-min measurements the blood glucose levels of T2DM group were significantly higher than those in the MET and CTRL groups (26.2 ± 4.1 mmol/L *vs*. 13.1 ± 2.5 mmol/L and 7.8 ± 1.4 mmol/L *p* < 0.001, respectively). The AUC values were significantly increased in the T2DM group (*p* < 0.001) and decreased in the MET group (*p* < 0.001). During the 1^st^ IPGTT, the AUC values were significantly higher in groups with diabetes. Although this difference was detected during the 2^nd^ IPGTT (*p* < 0.001), the AUC value in the T2DM group was higher than that in the MET group (*p* < 0.001).

**FIGURE 3:**
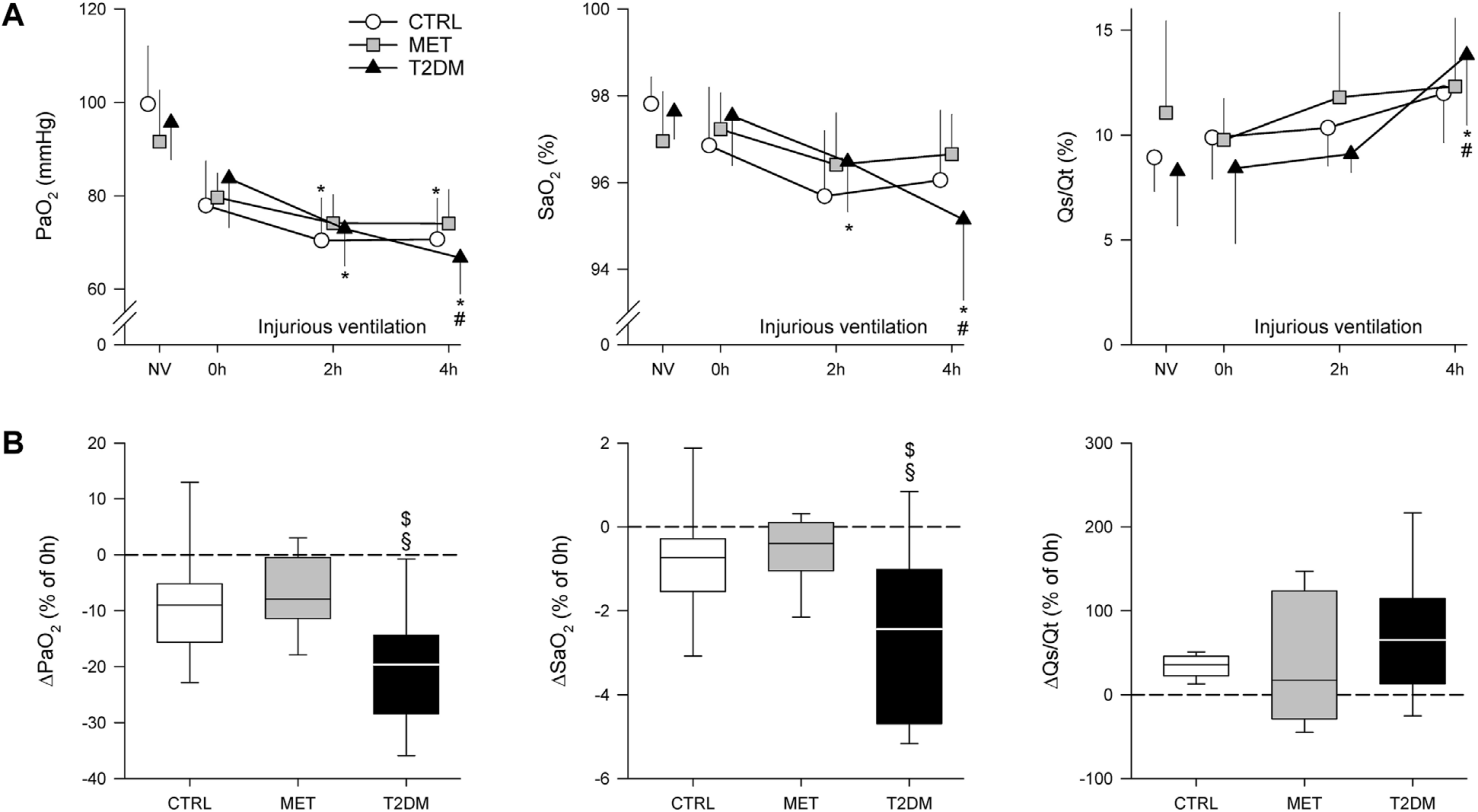
Partial oxygen pressure (PaO_2_) and oxygen saturation (SaO_2_) of the arterial blood and the intrapulmonary shunt fraction (Qs/Qt) obtained during volume-controlled ventilation with physiological parameters (NV) and injurious ventilation in control rats (CTRL), in rats with metformin-treated type 2 diabetes (MET), and in rats with untreated type 2 diabetes (T2DM). **(A)** *:* Absolute changes in mean values ± standard deviation (SD). **(B)** *:* Changes after 4 h of injurious ventilation values relative to the values obtained at 0 h. *p < 0.05 *vs*. 0 h; #*p* < 0.05 *vs*. 2 h; $*p* < 0.05 *vs*. CTRL; §*p* < 0.05 *vs*. MET.

Figure 3 shows the gas exchange parameters during normal ventilation (NV) and after injurious ventilation (0–4 h) in the study groups. Although PaO_2_ decreased in the CTRL group after 4 h of injurious ventilation (*p* < 0.05), this decline was more significantly manifested in the T2DM group (*p* < 0.001). Rats in the MET and CTRL groups demonstrated only tendencies for worsening in SaO_2_ and Qs/Qt, whereas rats in the T2DM group exhibited significantly compromised gas exchange due to injurious ventilation (*p* < 0.05). When the alterations in gas exchange indices were expressed as relative changes compared with the onset of injurious ventilation (0 h), significantly greater deterioration was observed in PaO_2_ and SaO_2_ in the T2DM group than in the other two groups (*p* < 0.05).

**FIGURE 4:**
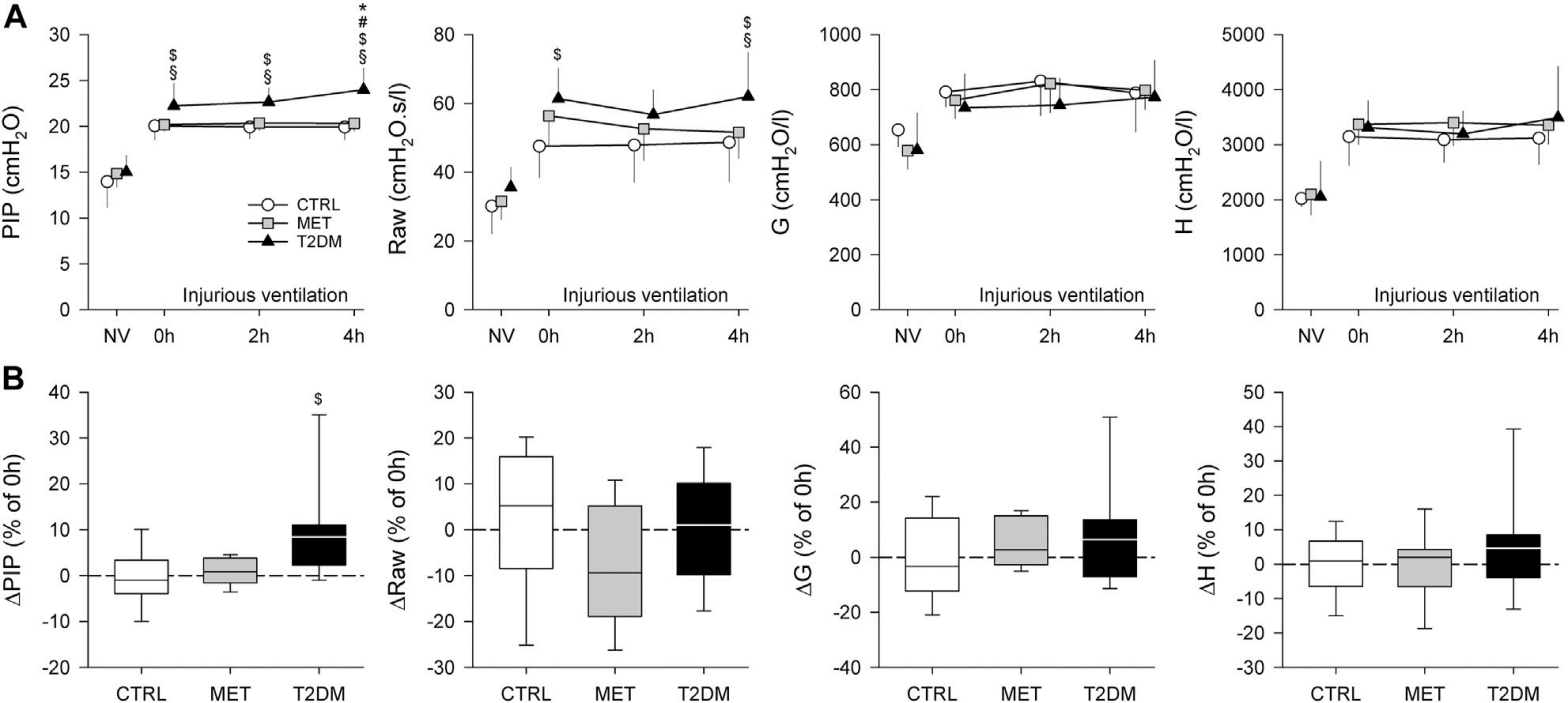
Peak inspiratory pressure (PIP), airway and viscoelastic respiratory tissue mechanical parameters obtained during volume-controlled ventilation with physiological parameters (NV) and injurious ventilation in control rats (CTRL), in rats with metformin-treated type 2 diabetes (MET), and in rats with untreated type 2 diabetes (T2DM). **(A)** *:* Absolute changes in mean values ±standard deviation (SD). **(B)** *:* Changes after 4 h of injurious ventilation values relative to the values obtained at 0 h. *p < 0.05 vs. 0 h; #*p* < 0.05 vs. 2 h; $*p* < 0.05 vs. CTRL; §*p* < 0.05 vs. MET. Abbreviations: PIP, Peak inspiratory pressure; Raw, airway resistance; G, tissue damping (resistance); H, tissue elastance.

Figure 4 depicts the changes in PIP and the airway and respiratory tissue parameters obtained during NV and injurious ventilation (0–4 h). Significantly higher PIP was recorded in the T2DM group than in the other study groups throughout the study period (*p* < 0.05), and the rats in the T2DM group exhibited significant increases in PIP after 4 h of injurious ventilation (*p* < 0.05). Rats in the T2DM group also showed higher Raw values than those in the CTRL group at the onset of injurious ventilation (0 h, *p* = 0.013). This difference was also observed after 4 h of injurious ventilation (*p* = 0.012), with significance also being detected in comparison with the MET group (*p* < 0.05). Respiratory tissue mechanical parameters exhibited no significant difference between the study groups at any time point of measurement.

**FIGURE 5:**
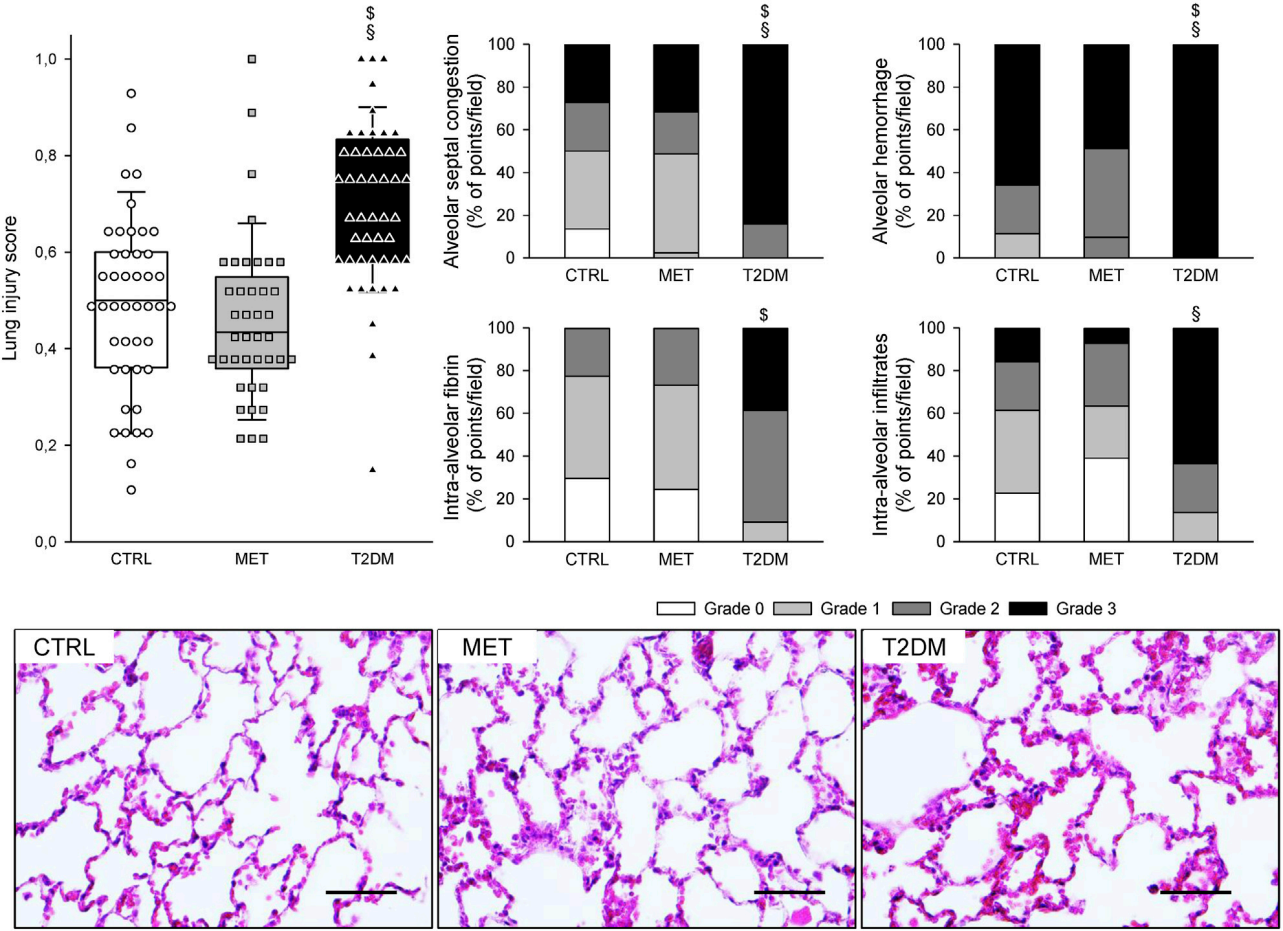
Lung injury score (LIS) in control rats (CTRL), in rats with metformin-treated type 2 diabetes (MET), and in rats with untreated type 2 diabetes (T2DM) and its components describing the different types of lung injuries. Each symbol represents the LIS value on a field of view. Representative images of lung sections stained with hematoxylin and eosin are also demonstrated (magnification × 400; scale bar: 50 µm). $*p* < 0.05 *vs*. CTRL; §*p* < 0.05 *vs*. MET.

Figure 5 summarizes the LIS in the three groups, describing the different injury types. LIS was significantly higher in the T2DM group than in the CTRL and MET groups (*p* < 0.001). Although the grades of intra-alveolar fibrin were significantly higher in the T2DM than in the CTRL group (*p* = 0.018), and the grades of intra-alveolar infiltrates were significantly higher in the T2DM group than in the MET group (*p* = 0.009), the alveolar septal congestion and alveolar hemorrhage were significantly more intense in the T2DM group than in both CTRL and MET groups (*p* < 0.001).

**FIGURE 6:**
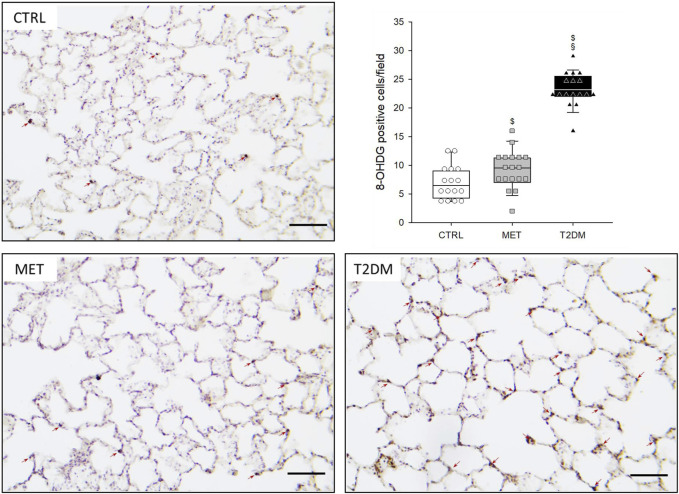
Representative lung sections of the three study groups stained with anti-8-OHDG immunohistochemistry. Red arrows indicate the 8-OHDG-positive cells (magnification × 200; scale bar: 100 µm). Abbreviations: CTRL, control group; MET, metformin-treated model of type 2 diabetes; T2DM, untreated model of type 2 diabetes. Graph represents the number of anti-8-OHDG positive-stained cells in the three study groups. $*p* < 0.05 *vs*. CTRL; §*p* < 0.05 *vs*. MET.

The number of anti-8-OHDG-positive-stained nuclei is presented in Figure 6. Rats in the MET group exhibited significantly higher number of positive-stained nuclei than those in the CTRL group (*p* = 0.037), whereas the number of positive-stained nuclei in the lungs of T2DM rats was significantly greater than that in both CTRL and MET rats (*p* < 0.001).

## Discussion

This study has revealed the potential of T2DM to worsen respiratory functions and lung injury following a biotrauma of prolonged mechanical ventilation according to decline in parameters reflecting gas exchange. Lung histological and immunochemical parameters also showed that diabetes enhanced VILI and oxidative DNA damage. Metformin treatment prevented the detrimental pulmonary consequences of long-term mechanical ventilation in the presence of diabetes.

In this study, a well-established model was adapted to induce T2DM by administering a single low dose of STZ to induce diffuse degeneration of pancreatic cells to imitate β-cell insufficiency combined with high-fat diet (Skovsø, 2014; Zhang and Hu, 2020). While the fasting glucose levels of the STZ-treated rats were not consistently above the threshold of 8.3 mmol/L, their blood glucose levels were significantly higher compared to the control animals. Furthermore, the 120-min blood glucose values were above the threshold of 11.1 mmol/L in all STZ-treated animals, while none of the control animals had abnormal blood glucose values. Consequently, according to the diagnostic criteria (Furman, 2015), rats in both STZ-treated groups (T2DM and MET groups) exhibited definitive blood glucose abnormalities characteristic to diabetes one week after the STZ treatment, before the initiation of metformin therapy. At the end of the pretreatment period, during the second tolerance test, the fasting and 120-min blood glucose values of the T2DM group exceeded both thresholds. The MET group showed a more controlled hyperglycemia during the second test, while the blood glucose curves of the CTRL animals were identical during both tolerance tests (Figure 2).

The translational animal model used in the present study aimed at mimicking the development of mild–moderate VILI in the absence of a preexisting pulmonary disorder. Accordingly, we applied no intervention to induce surfactant deficiency or proinflammatory treatments before initiating the prolonged mechanical ventilation. The alveolar overdistension and enhanced lung parenchymal shear stress as key features of VILI were generated by a combined application of high VT and low PEEP to facilitate the development of barotrauma, volutrauma, and atelectrauma in the rats of all experimental groups (Villar et al., 2009; Frank et al., 2008). Irrespective of the presence of T2DM, these mechanical stresses were manifested in deterioration in gas exchange (Figure 3) and in lung injury and oxidative DNA damage (Figures 5, 6). Interestingly, although a decline in the mechanical properties of the respiratory system due to the injurious ventilation was indicated by significant elevations in PIP in the T2DM group, the changes in the forced oscillatory parameters did not reach significance during the study period (Figure 4). This result can be explained by the application of excessively high VTs during the injurious ventilation (more than three times the normal) that assured maximal alveolar recruitment during the study period (Nieman et al., 2020). Moreover, the involvement of a significantly unaffected chest wall component in G and H may have blunted the sensitivity of these mechanical outcomes to detect mild–moderate lung injury (Südy et al., 2019). Conversely, markedly greater LIS and overexpression of 8-OHGD-positive cells in the lung tissue were observed in CTRL animals than those obtained in previous experiments in naïve rats without injurious ventilation (0.15 ± 0.12 *vs*. 0.49 ± 0.18 and 3.0 ± 1.0 *vs*. 7.0 ± 2.9, *p* < 0.05 for both) (Südy et al., 2020). These findings confirm the development of the structural and functional pathologies characteristic of VILI even in the control animals without metabolic disorder.

The most remarkable finding of the present study is the exaggeration of VILI in rats with untreated T2DM. The more severe detrimental consequences of untreated T2DM were evidenced by the greater magnitude of deteriorations in the gas exchange ability of the lungs and parameters reflecting Qs/Qt (Figure 3). As the respiratory mechanical parameters did not exhibit an excessive change in rats with diabetes, atelectatic lung volume loss was not likely to play a major role in the excessively compromised gas exchange (Figure 4). Alternatively, the exaggerated impairment of gas exchange in the diabetic animals can be attributed to the intrinsic lung tissue remodeling and inflammation with an impaired alveolar–capillary barrier, all leading to a reduced diffusion of gas molecules through the alveolar membrane. The involvement of this mechanism is confirmed by the histological findings evidencing alveolar septal congestion and hemorrhage associated with intra-alveolar deposition of fibrin and infiltrates (Figure 5). In accordance with our findings, previous studies have also demonstrated direct tissue damage in experimental models of VILI due to inflammation and oxidative damage of cellular components. These include oxidation of tissue lipid and protein components (Andrade et al., 2018; Fisher et al., 2021), increased levels of IL-1β, IL-6, IL-8, and TNF-α (Chen et al., 2018b). These molecular processes enhance the proinflammatory effects of prolonged hyperglycemia (Brownlee, 2001; Giacco and Brownlee, 2010), resulting in more severe lung injury.

A further noteworthy finding of this study is the ability of metformin to prevent the worsening of VILI subsequent to T2DM. The results of the 2^nd^ IPGTT performed after metformin treatments demonstrated the effectiveness of this therapy in leading to controlled hyperglycemia in the MET group through the following well-established mechanisms of action: inhibiting hepatic gluconeogenesis and reducing hepatic glucose output; increasing glucose uptake and utilization in peripheral tissues (muscle and fat); and improving energy metabolism in the muscle, fat, and liver through the activation of AMP-activated protein kinase (Foretz et al., 2010). It has been described that the AMP-activated protein kinase down-regulated inflammatory pathways such as the NF-κB pathway (Salminen et al., 2011), which might contribute to the beneficial respiratory effects of metformin. Consistent with previous results, metformin therapy had no significant effects on the baseline lung functional or structural parameters (Hitchings et al., 2016). However, the effects of this first-line antidiabetic therapy on the lungs were clearly manifested in the potential to prevent the T2DM-induced excessive worsening in gas exchange (Figure 3), the aggravation of lung injury (Figure 4), and the oxidative DNA damage (Figure 5). These findings suggest the ability of metformin therapy to not only reduce hyperglycemia and impaired glucose tolerance but also abolish the adverse pulmonary consequences of T2DM. Our results correspond to previous experimental and clinical findings demonstrating that adequate diabetes therapy prevents the development of lung injury as a complication of mechanical ventilation (Tsaknis et al., 2012; van den Berghe et al., 2001), although excessive hyperglycemia results in elevated expression of pro-inflammatory cytokines leading to severe lung injury (Bar-Or et al., 2019; Wu et al., 2020).

A few limitations relevant to the present study warrant consideration. Well-established models for treated and untreated T2DM were used in this study that reflect the pathogenesis of the human metabolic disease, including the chronic hyperglycemia, impaired glucose tolerance, and all consequential adverse pulmonary and systemic outcomes (Gheibi et al., 2017). However, the antidiabetic medication was applied through drinking water. Hence, rats in the MET group represent a patient population with high medication adherence, which represents only a fragment of patient population with treated diabetes (García-Pérez et al., 2013). A further technical aspect of this research protocol is the application of a relatively short injurious ventilation period (4 h). This time interval is equivalent to a far longer ventilation period in human subjects due to the significantly greater ventilation frequency in rats, and thus, such regimen has been used in numerous earlier studies to induce VILI in animals with healthy lungs (Villar et al., 2009; Wu et al., 2016). However, a more prolonged ventilation period may further augment the severity of VILI in the presence of T2DM, particularly if diabetes is associated with a chronic pulmonary disease, such as Chronic Obstructive Pulmonary Disease (Rogliani et al., 2015; Khateeb et al., 2019); these aspects may be subjects of further investigations. Our study evidenced an enhanced DNA damage after injurious ventilation in the presence of diabetes. However, evaluation of further biomarkers related to oxidative stress would be needed to confirm the role of this mechanism in pulmonary tissue damage and the exaggerated lung injury in the concordant presence of VILI and untreated T2DM.

## Conclusion

The results of the present study demonstrated that prolonged mechanical ventilation of diabetic lungs aggravates the functional and structural manifestations of mild–moderate VILI. Exaggerated lung injury resulting in more severe remodeling of the alveolar–capillary barrier is the primary cause of the declined gas exchange after sustained injurious mechanical ventilation. Although metformin therapy has no direct effect on lung function or gas exchange, our findings suggest that an adequate diabetes therapy with controlled hyperglycemia or euglycemia lowers the risk of developing ALI and, in severe cases, the development of ARDS subsequent to long-term mechanical ventilation.

## Data Availability

The raw data supporting the conclusions of this article will be made available by the authors, without undue reservation.

## Glossary

8-OHDG: 8-hydroxy-2ʹ-deoxyguanosine
ALI: acute lung injury
ANOVA: analyses of variances
AUC: area under the curve
CaO_2_: arterial oxygen content
CcO_2_: capillary oxygen content
CTRL: control
CvO_2_: venous oxygen content
DAB: diaminobenzidine
FOT: forced oscillations
G: respiratory tissue damping (resistance)
H: respiratory tissue elastance
IPGTT: intraperitoneal glucose tolerance test
LIS: lung injury score
MET: metformin
NF-κB: nuclear factor kappa
NV: normal ventilation
PaCO_2_: arterial partial pressure of carbon dioxide
PaO_2_: arterial partial pressure of oxygen
PEEP: positive end-expiratory pressure
PFA: paraformaldehyde
PIP: peak inspiratory pressure
Qs/Qt: intrapulmonary shunt fraction
Raw: airway resistance
SaO_2_: arterial oxygen saturation
SD: standard deviation
STZ: streptozotocin
T2DM: type 2 diabetes mellitus
VILI: ventilator-induced lung injury
VT: tidal volume
Zrs: input impedance of the total respiratory system
